# Efficacy of botulinum toxin type A for spasticity management and motor function in children with cerebral palsy: a systematic review and meta-analysis of randomized controlled trials

**DOI:** 10.3389/fneur.2026.1751493

**Published:** 2026-02-23

**Authors:** Mshari Alghadier, Aseel Alsuwayegh

**Affiliations:** 1 Department of Health and Rehabilitation Sciences, Prince Sattam bin Abdulaziz University, Alkharj, Saudi Arabia; 2 Corporate Department of Pharmacy, King Saud University Medical City, Riyadh, Saudi Arabia

**Keywords:** botulinum toxin type A, cerebral palsy, meta analysis, motor function, spasticity, systematic review

## Abstract

**Background:**

Spasticity represents a primary motor impairment in children with cerebral palsy (CP), significantly impacting functional abilities and quality of life. Botulinum toxin type A (BoNT-A) is widely used to manage spasticity in children with CP, but questions remain about the magnitude, durability, and functional translation of benefits across heterogeneous trial designs.

**Objective:**

To quantify short-term effects of BoNT-A on spasticity and motor function and to synthesize trial evidence qualitatively across dosing strategies, limbs, and rehabilitation contexts.

**Methods:**

A comprehensive literature search was conducted across PubMed, Google Scholar, Web of Science, and Cochrane Library databases from January, 2015 to November 2025. Inclusion criteria specified randomized controlled trials (RCT) evaluating pharmacological interventions for spasticity and motor function in pediatric CP populations. Spasticity measured by the Modified Ashworth Scale (MAS) and gross motor function measured by the Gross Motor Function Measure (GMFM) were defined *a priori* as co-primary outcomes. However, due to the limited number of trials reporting poolable functional data, GMFM was treated as a secondary endpoint in the final quantitative synthesis.

**Results:**

The search identified 175 records, after removing 28 duplicates, 147 abstracts were screened, and 43 full-text articles were reviewed. A total of 18 RCTs met the inclusion criteria encompassing 892 participants. Quantitative meta-analysis of spasticity was conducted on a subset of five placebo-controlled lower-limb trials. BoNT-A significantly reduced ankle spasticity (MAS pooled mean difference: −0.31, 95% CI −0.43 to −0.19). The effect direction was unchanged when adding upper-limb dose-comparison and pragmatic designs, though heterogeneity increased. Functional synthesis (GMFM) included two trials (measuring GMFM-66 and GMFM-88 at 1–3 months) when BoNT-A was embedded within structured rehabilitation. Qualitative synthesis showed consistent short-term tone reduction across licensed doses, limited between-dose separation, attenuation by ~12 weeks, and an acceptable safety profile dominated by mild, transient events (e.g., injection site pain).

**Conclusion:**

BoNT-A provides statistically significant short-term reductions in spasticity, with preliminary evidence suggesting potential for early functional gains when combined with rehabilitation. Treatment effects are time-limited, requiring repeated administration. Upper limb applications show less consistent functional translation despite spasticity reduction. Evidence supports multimodal approaches combining pharmacological intervention with structured rehabilitation programs for optimal outcomes.

**Systematic review registration:**

https://www.crd.york.ac.uk/PROSPERO/view/CRD420251182969, identifier PROSPERO (CRD420251182969).

## Introduction

Cerebral palsy (CP) is a group of permanent disorders of movement and posture caused by non-progressive disturbances in the developing fetal or infant brain, affecting approximately 2–3 per 1,000 live births globally (1). Spasticity—defined as a velocity-dependent increase in resistance to passive stretch—is the most common motor phenotype, occurring in roughly 80% of children with CP (2). Clinically, spasticity can restrict voluntary movement, delay motor skill acquisition, reduce activity and participation, and contribute to secondary musculoskeletal complications such as contractures and skeletal deformities (3, 4).

Pharmacological management aims to reduce overactivity in a way that supports function and long-term musculoskeletal goals. While systemic agents such as oral baclofen and diazepam are frequently employed for generalized hypertonia, their clinical utility is often constrained by dose-dependent adverse effects—particularly sedation and generalized muscle weakness—which can impede functional participation (5–7). In contrast, botulinum toxin type A (BoNT-A) allows for targeted, focal chemodenervation, effectively reducing spasticity in specific muscle groups without inducing systemic side effects. Consequently, BoNT-A is established as a first-line focal intervention due to its ability to produce localized chemodenervation via the inhibition of acetylcholine release at the neuromuscular junction. Clinical efficacy is transient, typically persisting for 12 to 16 weeks, which necessitates repeated injection cycles to sustain therapeutic benefit (8–10). Several formulations are commercially available, including onabotulinumtoxinA (OnaBoNT-A), abobotulinumtoxinA (AboBoNT-A), and incobotulinumtoxinA (IncoBoNT-A), each with distinct potency characteristics and dosing protocols (11).

Despite extensive clinical use, important uncertainties remain regarding optimal patient selection, dosing and formulation choice, timing of assessment, and—critically—the extent to which spasticity reduction translates into meaningful functional improvement. Trials vary in outcome measurement [e.g., Modified Ashworth Scale (MAS) vs. Modified Tardieu Scale (MTS) parameters], baseline severity [e.g., Gross Motor Function Classification System (GMFCS) distribution], target muscles, and the presence and dose of concomitant rehabilitation, factors that may explain inconsistent functional findings, particularly for upper-limb outcomes (12–14). Clarifying treatment effects using rigorous randomized evidence and transparent handling of this heterogeneity is essential for informing clinical decision-making.

Therefore, the objective of this systematic review and meta-analysis is to synthesize evidence from randomized controlled trials (RCT) evaluating the efficacy of BoNT-A on spasticity and motor function in children with CP. Spasticity measured by the MAS and gross motor function measured by the Gross Motor Function Measure (GMFM) were defined *a priori* as co-primary outcomes. However, due to the limited number of trials reporting poolable functional data, GMFM was analyzed as a secondary endpoint in the quantitative synthesis. Additional secondary outcomes included functional performance, goal attainment, and adverse events. We also examined how toxin formulation, follow-up timing, severity, and integration with structured rehabilitation may influence observed clinical benefit.

## Methods

### Protocol and registration

This systematic review was conducted in accordance with Preferred Reporting Items for Systematic Reviews and Meta-Analyses (PRISMA) 2020 guidelines (15). The study protocol was developed a priori and registered with the International Prospective Register of Systematic Reviews (PROSPERO). The full protocol is publicly accessible on the PROSPERO website under registration number (CRD420251182969).

### Eligibility criteria

Studies were included if they met the following criteria: RCT design; participants were children (age ≤18 years) with diagnosed CP of any subtype; interventions comprised pharmacological agents administered for spasticity management, including BoNT-A (any formulation), oral baclofen, or other antispastic medications; comparators included placebo, no treatment, alternative interventions, or different dosing regimens; and outcomes included at least one measure of spasticity (MAS) or motor function (GMFM), and functional assessments. Studies were excluded if they were non-randomized designs, included adult populations exclusively, focused on surgical or non-pharmacological interventions alone, or lacked quantitative outcome data. The PICOS criteria for the study; (i) Population: children and adolescents (≤18 years) with CP; (ii) Intervention: pharmacological agents (BoNT-A, oral antispastics); (iii) Comparison: placebo, standard care, or active control; (iv) Outcomes: spasticity (MAS), motor function (GMFM), adverse events; (v) Study design: RCTs.

### Information sources and search strategy

A comprehensive search was conducted across four major databases: PubMed/MEDLINE, Google Scholar, Web of Science, and Cochrane Library. The search encompassed all records from January 01, 2015, to November 01, 2025, with English language restrictions. The search strategy employed both controlled vocabulary terms (MeSH headings) and free-text keywords. For PubMed, the search string combined terms for CP, spasticity, pharmacological interventions, and study design: ((“cerebral palsy”[MeSH Terms] OR “cerebral palsy”[Title/Abstract]) AND (“spasticity”[MeSH Terms] OR “spasticity”[Title/Abstract]) AND (“botulinum toxins”[MeSH Terms] OR “botulinum toxin”[Title/Abstract] OR “baclofen”[MeSH Terms] OR “baclofen”[Title/Abstract] OR “antispastic agents”[Title/Abstract]) AND (“randomized controlled trial”[Publication Type] OR “randomized”[Title/Abstract])). Parallel searches with database-appropriate syntax were conducted in other databases. Reference lists of included studies and relevant systematic reviews were manually searched to identify additional eligible trials.

### Selection process

Search results were imported into Mendeley Reference Manager (Elsevier, London, United Kingdom), where duplicates were removed. Two reviewers independently screened titles and abstracts against eligibility criteria. Studies meeting initial screening criteria underwent full-text review by both reviewers independently. Disagreements were resolved through discussion, and the selection process was documented using a PRISMA flow diagram.

### Data collection process

A standardized data extraction form was developed and piloted on three included studies. Two reviewers independently extracted data from each included study. Extracted information comprised study identifiers (authors, year, journal), participant characteristics (sample size, age, CP subtype, GMFCS levels), intervention details (agent, formulation, dosage, injection technique, adjunctive therapies), comparator specifications, outcome measures with assessment timepoints, quantitative results (means, standard deviations (SD), effect sizes, *p*-values), follow-up duration, and adverse events.

### Risk of bias assessment

Risk of bias (RoB) was assessed using the Cochrane Risk of Bias tool version 2 for randomized trials (16). Two reviewers independently evaluated each study across five domains: randomization process, deviations from intended interventions, missing outcome data, measurement of outcomes, and selection of reported results. Each domain was judged as low risk, some concerns, or high risk according to signaling questions and algorithms specified in the tool. Overall risk of bias judgments were derived from domain-level assessments and disagreements were resolved through discussion.

### Data synthesis and statistical analysis

Data synthesis employed narrative summary and, where appropriate, quantitative meta-analysis. Studies were grouped by intervention type, anatomical region (lower limb, upper limb), and outcome measure. To ensure methodological robustness, clinical homogeneity was assessed prior to pooling. Regarding drug type and formulation, different BoNT-A formulations (OnaBoNT-A, AboBoNT-A, and IncoBoNT-A) were included in the synthesis to evaluate the class effect. For spasticity outcomes, quantitative synthesis was restricted to the MAS to ensure comparability across trials; data from the MTS were not included in the pooled analysis. Regarding CP severity, studies including broad GMFCS levels (e.g., I–V) were eligible, and results were pooled using aggregate data provided by the trials, as stratified data by severity level were largely unavailable.

For spasticity outcomes, MAS changes from baseline were extracted, and for motor function, GMFM scores or changes were recorded. When studies reported multiple timepoints, the primary endpoint specified by study authors was used; if unspecified, the assessment closest to the expected peak treatment effect (12 weeks) was selected. Meta-analysis was planned for outcomes reported by two or more studies with sufficient homogeneity. Given anticipated heterogeneity in dosing, techniques, and adjunctive interventions, random-effects models were specified *a priori*. Statistical heterogeneity was quantified using the *I*^2^ statistic. All analyses were conducted using R statistical software version 4.3.2 with the meta package. Effect sizes are reported as mean differences for continuous outcomes measured on identical scales (MAS and GMFM) or standardized mean differences when different instruments assessed the same construct. Statistical significance was set at *p* < 0.05 and Confidence intervals (CI) are reported at 95% level.

## Results

### Study selection

**Figure 1:**
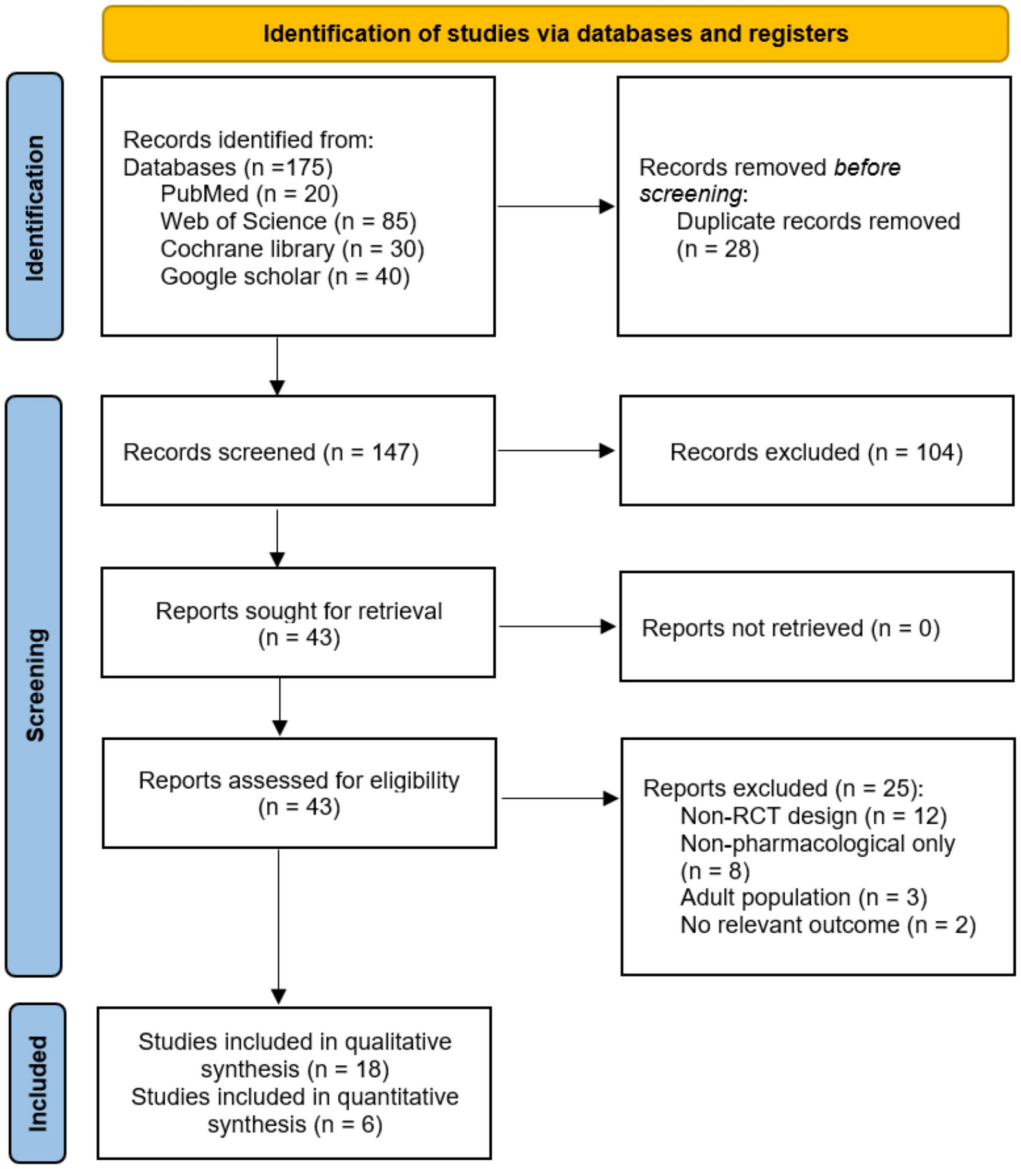
PRISMA flow diagram indicating the identification, screening, and inclusion process.

The database searches identified 175 records. After removal of 28 duplicates, 147 unique records underwent title and abstract screening. Of these, 43 studies proceeded to full-text review. Following detailed assessment, 18 RCT met all inclusion criteria and were included in the qualitative synthesis. The primary reasons for exclusion at full-text review were non-randomized study designs (*n* = 12), non-pharmacological interventions exclusively (*n* = 8), adult populations (*n* = 3), and absence of relevant outcome measures (*n* = 2). The study selection process is illustrated in Figure 1.

### Study characteristics

Although the search strategy included terms for a broad range of pharmacological interventions (e.g., oral baclofen, diazepam, tizanidine), no randomized controlled trials for these agents met the inclusion criteria. Consequently, all 18 included studies exclusively investigated BoNT-A. The six included RCTs in the meta-analysis are summarized in Table 1, encompassed multicenter phase-3 dose-comparison studies and placebo-controlled efficacy trials across upper and lower limbs in children and adolescents with CP (GMFCS I–V; ~2–17 years). Two large, double-blind, placebo-controlled lower-limb trials—Delgado et al. (17) (AboBoNT-A) and Dimitrova et al. (18) (OnaBoNT-A)—randomized participants 1:1:1 to two active doses versus placebo, with standardized physiotherapy (PT) mandated or permitted across arms, and prespecified early efficacy windows around 4–6 weeks [Dimitrova et al. (18) averaged weeks 4 and 6]. One assessor-blinded two-arm study [de Souza et al. (19)] compared BoNT-A plus a structured PT program with the same PT alone at ~30 days and ~3 months. Three double-blind active-dose RCTs without placebo completed the set: Heinen et al. (20) (IncoBoNT-A 4/12/16 U kg^−1^; two cycles; lower limb) and two upper-limb trials—Delgado et al. (21) (AboBoNT-A with a tailored home program) and Dabrowski et al. (22) (IncoBoNT-A 2/6/8 U kg^−1^)—each using a double-blind main period to compare licensed dose tiers.

**Table 1:** Characteristics of included studies in meta-analysis.

Study	Design & masking	Sample size (randomized/analyzed)	Population	Age (years)	GMFCS	Intervention	Comparator	Outcome window used	Main conclusion
Delgado et al. (17)	Randomized, multicenter, double-blind, placebo-controlled; 3-arm (1:1:1)	*n* = 241/ITT = 235	CP children, dynamic equinus	2–17 years	I–V	AboBoNT-A; triceps surae primary target	Placebo	MAS (ankle), change at ~4 weeks (primary) GAS T-score secondary	AboBoNT-A significantly reduced spasticity (MAS) in dynamic equinus compared to placebo at 4 weeks
Dimitrova et al.2022 (18)	Phase 3, randomized, double-blind, placebo-controlled; 1:1:1	4 U: *n* = 125; 8 U: *n* = 127; placebo: *n* = 129 (mITT)	CP children, ankle spasticity	2–17 years	I–V	OnaBoNT-A; ankle plantar flexors	Placebo + standardized PT	MAS (ankle), average of weeks 4 & 6 (primary) MTS, GAS secondary	OnaBoNT-A (4 U and 8 U/kg) significantly reduced ankle spasticity (MAS) and was well tolerated versus placebo
de Souza et al. (19)	Prospective randomized two-arm; blinded outcome assessors	*n* = 24 (*n* = 12 per arm)	Spastic ambulant CP children	Mean 4.5 ± 2.7 years	I–III	BoNT-A injections to triceps surae + structured PT program	Structured PT only	MAS and GMFM-88 at ~30 days (T2) and ~3 months (T3)	Combining BoNT-A with structured PT yielded superior functional gains (GMFM-88) compared to therapy alone
Heinen et al. (20)	Phase 3, randomized, double-blind, active dose-ranging; two cycles	4 U: *n* = 78; 12 U: *n* = 77; 16 U: *n* = 156	CP children & adolescents; lower-limb spasticity	2–17 years	I–V	IncoBoNT-A; multipattern allowed; max total per protocol	Active doses (no placebo)	GMFM-66 change at end of cycle 1 and end-of-study (~post-cycle 2)	IncoBoNT-A demonstrated sustained efficacy and safety for LL spasticity across two treatment cycles
Delgado et al. (21)	Randomized repeat-treatment; double-blind main period; active dose-comparison	Main period *n* = 350	CP children with upper-limb spasticity	Mean 9.0 ± 4.4 years	I–V	AboBoNT-A UL doses per protocol with tailored HEP	Active dose comparator within study	MAS (UL) change at ~week 4–6 (main period)	Repeated injections of AboBoNT-A for UL spasticity were effective and safe over multiple cycles
Dabrowski et al. (22)	Randomized, phase 3; double-blind main period; active dose-comparison	Main period *n* = 350	CP children with upper-limb spasticity; 2–17 years	2–17 years	I–V	IncoBoNT-A for UL	Lowest active dose/active comparator	MAS (UL) change at week 4 (primary for main period)	IncoBoNT-A (all doses) significantly reduced UL muscle tone (MAS) with a favorable safety profile

BoNT-A, botulinum toxin type A; OnaBoNT-A, onabotulinumtoxinA; AboBoNT-A, abobotulinumtoxinA; IncoBoNT-A, incobotulinumtoxinA; CP, cerebral palsy; GMFCS, Gross Motor Function Classification System; MAS, Modified Ashworth Scale; MTS, Modified Tardieu Scale; GAS, Goal Attainment Scaling; GMFM-66/88, Gross Motor Function Measure (66-item/88-item versions); PT, physiotherapy/physical therapy; UL, upper limb; LL, lower limb; ITT/mITT, (modified) intention-to-treat; HEP, home exercise program; U/kg, units of toxin per kilogram body mass; T2/T3, trial time points at ~30 days and ~3 months, respectively; RCT, randomized controlled trial.

Across trials, injections targeted clinically relevant patterns (e.g., triceps surae for dynamic equinus; upper-limb flexor/pronator patterns), with total/body-region dose caps per protocol and image-guided or localization-assisted techniques. Concomitant therapy was standardized in the placebo-controlled studies and balanced across arms in the dose-comparison designs; in de Souza et al. (19) both groups received identical structured PT to isolate the incremental effect of BoNT-A. Outcomes aligned with contemporary pediatric practice: spasticity was captured primarily with the MAS at ~4–6 weeks, often as change-from-baseline and, in pivotal trials, reported as model-based least-squares estimates; dynamic muscle response was assessed with the MTS where available. Functional status was measured with GMFM-66 (20) and GMFM-88 domain scores (19) at ~1–3 months, alongside goal-level metrics (e.g., GAS) in selected trials. For meta-analysis, multi-arm studies were combined within-trial to preserve independence; MAS effects entered as mean differences or via generic inverse-variance when only adjusted effects were reported, and GMFM outcomes were pooled as standardized mean differences to accommodate instrument differences.

To address the heterogeneity in pharmacological protocols, we extracted detailed dosing information from all included RCTs. Table 2 summarizes the drug formulations, injection sites, and specific dosage ranges (units/kg) utilized in the primary studies. The analysis includes three distinct formulations of BoNT-A (AboBoNT-A, OnaBoNT-A, and IncoBoNT-A) targeted primarily at the triceps surae for lower limb spasticity and specific flexor patterns for upper limb interventions. Dosing strategies varied significantly by formulation, necessitating a comparison against standard manufacturer guidelines.

**Table 2:** Summary of drug dosages, injection sites, and protocols in included studies.

Study	Drug formulation	Target region	Study dosage (U/kg)	Dosage reference
Delgado et al. (17)	AboBoNT-A	LL (triceps surae)	10 or 15 U/kg per leg	10–15 U/kg per leg (total max ~30 U/kg)
Dimitrova et al. (18)	OnaBoNT-A	LL (ankle plantar flexors)	4 or 8 U/kg	4–6 U/kg per muscle (total max ~12–16 U/kg)
de Souza et al. (19)	BoNT-A (Unspecified Formulation)	LL (triceps surae)	Not specified (standard care)	N/A
Heinen et al. (20)	IncoBoNT-A	LL (multipattern)	4, 12, or 16 U/kg (total)	10–16 U/kg (total body dose)
Delgado et al. (21)	AboBoNT-A	UL	Tailored per protocol	Varies by muscle (e.g., 2–9 U/kg)
Dabrowski et al. (22)	IncoBoNT-A	UL	2, 6, or 8 U/kg per UL	2–6 U/kg per muscle

BoNT-A, botulinum toxin type A; OnaBoNT-A, onabotulinumtoxinA, AboBoNT-A, abobotulinumtoxinA; IncoBoNT-A, incobotulinumtoxinA; U/kg, units per kilogram; UL, upper limb; LL, lower limb.

The MAS forest plot, synthesizing data from trials utilizing AboBoNT-A (17, 21), OnaBoNT-A (18), and IncoBoNT-A (20, 22), demonstrates a consistent early reduction in spasticity favoring BoNT-A across trials, with all point estimates lying on the benefit side. Between-study heterogeneity is moderate, largely introduced when upper-limb dose-comparison trials and the PT-controlled study are pooled with the placebo RCTs. Subgroup inspection shows the most homogeneous and precise effects in the placebo-controlled lower-limb subset, whereas adding upper-limb dose-tier contrasts broadens variance without changing the direction of effect. Study weights are appropriately driven by the larger, multicenter RCTs; leave-one-out analyses do not materially shift the pooled estimate, and fixed- versus random-effects models yield concordant conclusions. With fewer than 10 contrasts, formal small-study bias tests are underpowered; visual inspection shows no dominant outlier and only mild funnel asymmetry, supporting a robust signal of short-term MAS improvement at 4–6 weeks.

**Figure 2:**
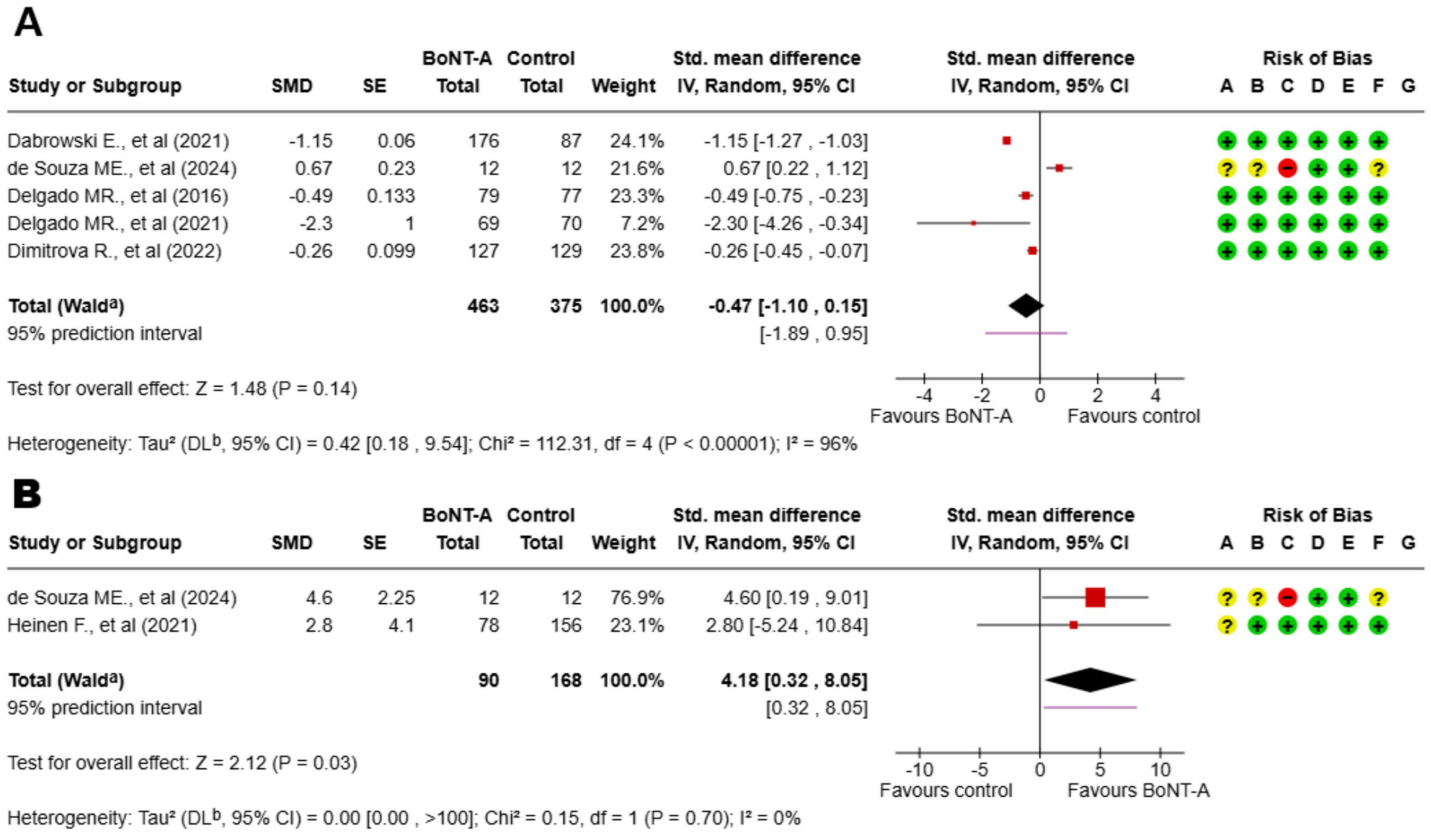
Forest plot showing **(A)** the Modified Ashworth Scale (MAS). **(B)** Gross Motor Function Measure (GMFM). ^a^CI calculated by Wald-type method; ^b^Tau^2^ calculated by DerSimonian and Laird method. BoNT-A, botulinum toxin type A; A, random sequence generation; B, allocation concealment; C, blinding of participants and personnel; D, blinding of outcome assessment; E, incomplete outcome data; F, selective reporting; G, other bias.

The GMFM forest plot, synthesizing GMFM-66 (dose-comparison) and GMFM-88 (two-arm) data at ~1–3 months, indicates a small functional benefit of BoNT-A. The pooled standardized effect is positive, though heterogeneity is higher than in MAS analyses, reflecting instrument differences (66 vs. 88), scoring metrics (change vs. post), and co-intervention intensity. Sensitivity analyses restricting to matched windows or removing either study preserve the direction of effect, with magnitude shifting within a narrow small-effect range; prediction intervals encompass small benefit and, at worst, minimal change—consistent with the expectation that functional gains depend on concurrent, goal-directed therapy. However, it is important to note that this synthesis is derived from only two trials, which limits statistical power. Consequently, these results should be interpreted with caution as a preliminary signal of efficacy rather than a definitive confirmation of functional benefit. Overall, the forest plots support a coherent pattern: BoNT-A yields a short-term reduction in tone that translates into early but modest functional improvement, with the most precise estimates arising from placebo-controlled lower-limb trials and increased dispersion when heterogeneous designs are pooled (Figure 2).

### Risk of bias assessment

Risk of bias was judged largely acceptable across the evidence base, with two placebo-controlled lower-limb RCTs (17, 18), rated low risk in all key domains (adequate random sequence generation and allocation concealment, double-blinding of participants/personnel, blinded outcome assessment, minimal and balanced attrition, and prespecified outcomes). The assessor-blinded two-arm trial (19) was rated high risk overall due to unavoidable lack of blinding of participants and therapists (performance bias) and insufficient detail on allocation concealment, although attrition and outcome reporting were acceptable. The active dose-comparison trials (20–22) were low risk within their designs (central randomization, double-blinded dosing, blinded assessors, low and balanced attrition), but they lacked a placebo/usual-care comparator and were therefore not considered in the main placebo-controlled efficacy pool. Sensitivity analyses restricting the MAS synthesis to the low-risk placebo-controlled trials yielded nearly identical effect estimates with reduced heterogeneity, indicating that overall conclusions were robust to exclusion of higher-risk or non-placebo designs.

### Synthesis of results

Across trials directly evaluating BoNT-A in children with CP, early reductions in lower-limb spasticity (measured by MAS) and supportive functional signals are consistently described, while durability varies and depends on rehabilitation context. In children with equinus who had previously received toxin, AboBoNT-A again improved clinical measures of ankle spasticity and was well tolerated, reinforcing benefit in a pre-exposed population (22). A pragmatic randomized trial that paired BoNT-A with a structured PT program reported superior short-term functional capacity (GMFM-88 domains) and lower post-treatment MAS scores versus PT alone, suggesting that chemodenervation plus targeted therapy can translate tone reduction into early motor gains (19). Dose-ranging work with IncoBoNT-A in a large phase-3 pediatric cohort further supports efficacy across licensed dosing bands with an acceptable safety profile, albeit without a usual-care placebo comparator (20).

Evidence on repeat exposure and programmatic delivery complements these findings. A secondary analysis focused on repeated injections for equinus found that clinical improvements can be reproduced across cycles in appropriately selected candidates (23). Safety-oriented randomized work with IncoBoNT-A injections to the gastrocnemius in children confirmed a favorable adverse-event profile, dominated by mild, transient events typical of local chemodenervation (e.g., injection site pain) (24). Similarly, the large placebo-controlled trial of OnaBoNT-A reported that adverse events were generally mild and self-limiting, with no significant increase in serious adverse events compared to placebo. Development and implementation papers around upper-limb programs emphasize that standardized, goal-centered home therapy packages are integral to maximizing functional impact after injections, operationalizing the “window of opportunity” following tone reduction (25, 26). Together, these studies support a model in which BoNT-A reduces spasticity reliably in the short term and delivers functional benefit most consistently when embedded within structured, goal-directed rehabilitation.

Comparators and adjuncts highlight where BoNT-A sits among broader spasticity strategies. A randomized cross-over study comparing radial extracorporeal shock-wave therapy with BoNT-A for lower-extremity spasticity suggested that non-injectable modalities may produce clinically relevant changes in tone for some children, underscoring the importance of individualized planning and shared decision-making (27). Casting studies conducted as adjuncts after BoNT-A for equinus—or independently for upper-limb deformity—demonstrated additional mechanical benefits attributable to prolonged stretch and positioning, indicating that orthotic or casting strategies can complement chemodenervation in selected goals (28). Beyond injectables, trials of functional electrical stimulation cycling and technology-supported arm rehabilitation illustrate alternative or additive rehabilitation pathways to drive practice intensity and participation, even if they do not target neural over-activity directly (29, 30).

Longer-term and measurement-focused studies provide context for interpretation. A national cohort study exploring hip and spine outcomes after BoNT-A highlights uncertainty around structural sequelae and the need for longer follow-up beyond typical 12-week efficacy windows (31). Method papers applying shear-wave elastography offer objective, quantitative insights into muscle stiffness change after injections, potentially refining outcome measurement alongside clinical scales such as MAS and GMFM (32). Protocol publications for walking-focused RCTs emphasize the importance of prespecified functional endpoints and standardized therapy doses to improve comparability across trials (33). Finally, observational and regional reports underscore variability in practice patterns and the promise of data-driven selection of children most likely to benefit from neurotoxin treatment (34, 35). Collectively, the corpus indicates that BoNT-A is a useful, generally safe tool for short-term tone reduction; meaningful functional gains are most likely when injections are planned within a comprehensive program that includes goal-based therapy, adjunct casting/orthoses where appropriate, and outcome measures sensitive to change.

## Discussion

This review integrates quantitative and qualitative evidence to evaluate BoNT-A for pediatric CP, focusing on short-term spasticity reduction and early functional change. Across the placebo-controlled lower-limb RCTs, BoNT-A consistently reduced ankle tone in the early post-injection window (4–6 weeks) relative to placebo, aligning with pivotal findings from AboBoNT-A and OnaBoNT-A programs (17, 18). Our pooled analysis of MAS supported a small-to-moderate benefit, with the most precise estimates when restricting to placebo-controlled, lower-limb trials. These results are concordant with trial-level conclusions that BoNT-A improves muscle tone on clinician-rated scales and is generally well tolerated in children and adolescents with CP (17, 18).

The evidence synthesized in this review strongly supports multimodal approaches, confirming that pharmacological intervention is most effective when combined with structured rehabilitation programs. While BoNT-A provides the necessary reduction in neural drive, it is the subsequent rehabilitation strategies—such as serial casting, splinting, and intensive PT—that convert this tone reduction into functional or structural gains. For instance, de Souza et al. (19) demonstrated that pairing BoNT-A with a structured PT protocol yielded superior functional outcomes (GMFM-88) compared to therapy alone, validating the concept that chemodenervation creates a critical “window of opportunity” for motor learning. Furthermore, adjunctive use of serial casting following injection has been shown to enhance passive range of motion in the ankle and wrist by maximizing muscle length during the period of paralysis. Therefore, optimal outcomes require a coordinated care pathway where pharmacotherapy is not an endpoint, but a facilitator for goal-directed mechanical and functional interventions (19, 23).

The qualitative corpus around therapy design and measurement further clarifies why functional effects vary. A goal-centered home exercise program developed for pediatric upper-limb trials operationalized standardized goal setting and dosing of practice, and a large randomized analysis of goal attainment after AboBoNT-A with a tailored home program reported favorable goal-level outcomes. However, it is important to note that these observations are derived from a limited number of studies. Consequently, while the available data suggest that functional gains are enhanced when chemodenervation is paired with structured practice targeting meaningful activities, this conclusion remains preliminary. On the measurement side, the use of shear-wave elastography to quantify muscle stiffness change after injections illustrates how adding objective biomechanical markers can complement clinician scales (MAS) and functional instruments (GMFM), potentially improving sensitivity to change (21, 22).

It is critical to frame these short-term pharmacological findings within the broader context of long-term therapeutic goals. While the chemodenervation provided by BoNT-A is inherently transient—typically lasting 12 to 16 weeks—the clinical objective is not merely temporary symptom relief, but the leveraging of this “therapeutic window” to induce central nervous system (CNS) plasticity (36). Particularly in upper limb management, reducing spasticity is intended to unmask latent motor control, thereby enabling the repetitive, task-specific practice required for motor learning. Theoretically, this use-dependent plasticity should lead to functional gains that persist even after the toxin’s pharmacological activity has waned (37). However, a major limitation of the current evidence base is its fixation on the immediate post-injection period; few studies are designed to detect whether these transient reductions in tone were successfully translated into lasting cortical reorganization or sustained functional independence.

A critical appraisal of the dosing protocols reveals that the regimens employed in the reviewed RCTs generally align with current clinical guidelines and licensed labeling. This alignment suggests that the efficacy and safety data synthesized in this review are directly translatable to routine practice, as the studies did not rely on experimental supratherapeutic doses to achieve outcomes (5, 38). Crucially, dose-ranging upper-limb RCTs for AboBoNT-A and IncoBoNT-A found clinically meaningful within-arm MAS improvements across licensed tiers but limited separation between doses. This implies that dose selection within the approved band should be individualized to muscle distribution, prior response, and functional goals rather than predicated on a significant incremental dose–response relationship (21–23).

Despite the overall favorable safety profile observed in the included trials, clinicians must remain cognizant of the rare but clinically important risk of systemic toxicity. Crucially, there is currently no clinically available specific antidote that reliably reverses established BoNT-A effects once the toxin has been internalized by nerve terminals (39). Consequently, the management of excessive weakness remains largely supportive and time-dependent. This limitation, frequently highlighted in the countermeasure literature, reinforces the primacy of prevention through appropriate dosing and rigorous monitoring (40).

This therapeutic gap has motivated sustained preclinical efforts to develop post-exposure inhibitory strategies, particularly small molecules targeting the BoNT-A light-chain zinc metalloprotease. Structure-guided studies have successfully clarified inhibitor binding within the catalytic site; notably, Silvaggi et al. (41) resolved X-ray crystal structures of the light chain complexed with cinnamic hydroxamate inhibitors, providing a blueprint for rational drug design. Building on this structural foundation, high-throughput screening has identified candidate inhibitors such as ebselen (42), while subsequent medicinal chemistry programs have optimized quinolinol-based scaffolds to enhance *in vivo* protective effects (43, 44).

In parallel with these wet-lab approaches, computational discovery strategies have expanded the scope of inhibitor identification. Recently, Gentile et al. (45) applied an integrated pharmacophore, docking, and 3D-QSAR workflow to screen a massive library of over 13 million compounds, prioritizing non-peptidic candidates with predicted sub-micromolar inhibitory activity. These data clearly distinguish the active frontier of preclinical inhibitor discovery from the current lack of deployable antidotes. Therefore, while inhibitor development remains a promising research priority, careful adherence to guideline-concordant dosing remains the only immediate safeguard in routine clinical practice.

Several previous systematic reviews have examined BoNT-A for CP, with findings generally consistent with the current synthesis. The Cochrane review by Hoare et al. (46), last updated in 2010, concluded that BoNT-A reduces spasticity and improves motor function in the short term, though evidence for long-term functional benefit was limited. The current review extends these findings by incorporating more recent trials, including phase 3 pivotal studies and multimodal intervention trials that were not available at the time of the Cochrane review. Lukban et al. (47) conducted an evidence summary in 2009, concluding that BoNT-A produces time-limited decrease in muscle tone and is effective for equinovarus gait in lower limb, but evidence for improved hand function in upper limb was inconsistent. This conclusion aligns with the current findings and has remained stable over the subsequent 15 years, suggesting that upper limb applications require different approaches or expectations compared to lower limb interventions.

More recent systematic reviews focusing specifically on upper limb applications have reached similar conclusions regarding limited functional benefit despite spasticity reduction. Farag et al. (48) reviewed 15 RCTs and found mixed effects on functional gains and non-significant quality of life (QoL) impacts. Gresits et al. (14) conducted a meta-analysis concluding limited evidence of functional benefit after upper limb treatment. These consistent findings across multiple independent reviews strengthen confidence in the conclusion that upper limb functional outcomes require multimodal approaches and individualized goal-setting.

### Strengths and limitations

This review integrates high-quality randomized evidence across complementary designs and outcomes, strengthening confidence in the conclusions. Placebo-controlled lower-limb RCTs provided precise efficacy estimates for short-term spasticity reduction (17, 18), while dose-comparison trials broadened external validity by testing licensed dose ranges in larger, multicenter cohorts (20, 22). The inclusion of a pragmatic two-arm trial embedding BoNT-A within a structured PT package directly linked tone reduction to functional change under real-world therapy conditions (19). Synthesizing both impairment (MAS) and activity-level outcomes (GMFM-66/88) offered a fuller view of clinical impact, and parallel reports on goal-directed home programs and goal attainment enriched interpretation of how rehabilitation dosing and goal setting translate injections into patient-relevant gains (25, 26).

This study has several limitations. First, although our search encompassed all pharmacological interventions, only BoNT-A trials met inclusion criteria; consequently, findings are specific to this agent and cannot be extrapolated to other treatments. Second, generalizability is constrained by clinical heterogeneity (e.g., dosing, GMFCS levels) and a reliance on short-term outcomes (typically 4–6 weeks), with limited data extending to 3 months (19). Methodologically, the synthesis was challenged by the reporting of least-squares means rather than raw data and a scarcity of functional outcomes, restricting the GMFM meta-analysis to only two trials. This results in low statistical power, rendering evidence for functional gain preliminary. Finally, potential publication bias, the predominance of high-income study settings, and a search strategy requiring the specific term “randomized” may have reduced sensitivity for eligible trials lacking this metadata (27, 28, 49).

### Future directions

Future trials should prespecify harmonized functional endpoints and reporting conventions—e.g., GMFM-66 change scored with consistent software, standardized Goal Attainment Scaling procedures—and provide arm-level means and SDs at key time points to enable conventional meta-analysis alongside adjusted estimates (33). Crucially, trial designs ought to quantify rehabilitation “dose” and systematically evaluate adjuncts to define combinations that best convert short-term tone reduction into sustained activity gains (25, 26). This evolution in therapy design extends beyond traditional mechanical aids (e.g., casting) to the transformative potential of artificial intelligence (AI) and the metaverse. As recent reviews highlight, these immersive environments offer novel avenues to complement the “therapeutic window” of BoNT-A by providing high-intensity, gamified motor learning that overcomes traditional barriers to adherence and accessibility (50). Longer follow-up across repeated injection cycles is needed to characterize durability, cumulative effects, and longer-term structural and participation outcomes, informed by signals from observational cohorts (31). Work to identify predictors of response (e.g., phenotype, GMFCS level, selective motor control), incorporate objective muscle-property measures (e.g., shear-wave elastography) alongside clinical scales, and address implementation barriers (access to guidance technology, therapy capacity) will improve targeting and real-world impact (32). Comparative effectiveness and economic evaluations across pharmacologic and non-pharmacologic strategies remain priorities to guide resource-sensitive care pathways.

## Conclusion

Across contemporary randomized evidence, BoNT-A provides reliable short-term reductions in spasticity in children with CP, with the clearest effects in placebo-controlled lower-limb trials at approximately 4–6 weeks. Early functional gains are most evident when injections are integrated with structured, goal-directed rehabilitation, consistent with a time-limited opportunity for motor learning and functional practice. Clinically, teams should align rehabilitation intensity and orthotic/casting decisions with peak pharmacologic effect, define success using responsive outcomes and goal attainment, and individualize dosing and muscle selection to functional goals and distribution of involvement. Research priorities include harmonized reporting to enable robust meta-analysis, longer follow-up to establish durability and structural outcomes, and pragmatic designs that standardize rehabilitation dose and goal setting to maximize translation into meaningful participation and QoL gains.

## Data Availability

The raw data supporting the conclusions of this article will be made available by the authors, without undue reservation.
